# Empirical Publications

**Published:** 1856-01

**Authors:** 


### EDITORIAL DEPARTMENT.

The following sensible remarks we copy from that excellent periodical, the *Western Lancet*, and commend them to the consideration of the profession, especially the medical journalists of the country:

"But, notwithstanding there is much impurity mingled in the great current of our periodical medical literature, it is generally not infectious, and leads to no serious contamination of the whole, for it is thrown in with an honest purpose, but without mature reflection or experience. There is, however, a side current coming from the most loathsome, marshy, miasmatic districts, that, we are sorry to find, is throwing masses of putrid filth into the main stream. We allude to the reports of cases that never occurred, to long articles describing discoveries that were never made, and to the publication of books that have nothing in them but what is stolen bodily from works of the regular profession, and a few specimens of the foul secretion of distempered brains. They are the productions of a set of men who flourish in Cincinnati—who are not acknowledged by the regular profession—who live in open violation of the code of ethics—who slander, abuse, vilify, and cheat the regular profession in every way they can devise—who are themselves the very dregs of society, and only acquire notoriety by their plagiarisms—their buncombe writings and buncombe reports of cases that they so industriously produce.

Now these productions would cause no very great harm to the regular profession, and very little benefit their authors, if the editors of the regular medical journals would not admit them to their pages, or give them flattering notices or reviews. At home, where their cunning is understood, and their designs fully appreciated, the productions of these men are not offered to the journals, nor are their books sent to them for review, but it is to editors of the east—the learned, the enlightened east—that they are sent, and anon the physicians of Cincinnati are mortified and humiliated at finding printed, in the most highly prized journals they receive, articles which they could not be forced to accept, much less read, if sent to them through any other channel. But the injury and injustice done the regular profession by this practice does not end in simple mortification—the trick is now prepared, and its greatest power for doing evil is to be displayed. These fellows claim that they are persecuted by the regular profession at home with malice and envy, and thus gain some sympathy from the ignorant portion of the community. They readily prove that they are in good fellowship with the regular profession abroad, by drawing from their pockets an eastern or northern journal of well known reputation, and showing that their articles are there printed, and that their books and charts are there noticed and eulogised."

We are compelled to admit there is too much of laxness and indifference manifested for the dignity of the medical profession by its own membership. Quackery, one of those evils which crawled its beslimed length out of Pandora's box to the injury of the human family, may continue to drag itself uninterruptedly along and coil around us and yet we listen calmly to its hissing, witness its springs, and observe unconcernedly, the wide diffusion of its poison. Some who hold prominent places in the profession, maintain that it is not to be destroyed by pursuing it,—others that these evils grow and multiply by proscribing them, —and others that we have serpents enough among ourselves, equally poisonous and deadly, with those moving around and just without us.

There is some semblance of reason in all this, and yet it is only a *show* of plausibility. It is not true in fact. Apply the same rule to other abuses and all reforms will cease. Reformation presumes an existing wrong, and the very first step in reform is to expose the error, that it may be perceived and known and when known, to be avoided. But the necessity of the reform lies in the magnitude of the error, and no one would estimate the magnitude of the evil unless exposed. This is the experience of all the great reforms, whether in religion, morals, or politics.

Because quackery exists, a sophistical mode of reasoning leads to the conclusion, most unwise and unsound indeed, that the efforts to restrain it have been wholly unavailing. Who can say assuredly, that it has not been kept at bay by the public exposures which have been made of it, and the attacks from time to time upon its strongholds? Who can say with assurance, that, but for this continued warfare, it would not have by this time acquired a monster power? Take the converse of the proposition, and admit for the sake of argument, that persecution has breathed vitality into it; that it was developed under its germinal influences, and grown by the nutrition subsequently and continually derived from it. If opposition has proved to be its *pabulum*, the necessary and legitimate inference would be that, had it not been furnished with this nutrition, it would not have existed, and who will venture to assert or prove this? The course then, in order to stifle it, would be to treat it temperately, kindly, yea *fondly*, and it must die. To destroy vice in our midst, from this showing, would be to encourage it, to *smile* upon it, and to furnish it the means of growth and spread, in order that it may destroy itself. To destroy rank weeds, agreeably to this logic, they must be cultivated. It is unreasonable, and we have no patience with such logic. For our part, we do not relish this squeamishness, this sensitive cautiousness, this nice, over-strained course of policy, and this nicely adjusted conservatism. We are not of that class who will hesitate to speak the truth, lest it might offend a nice *somebody*, nor to expose error, corruption, and wrong, lest we bring anathemas and curses upon our head. We would rather be engaged in the advocacy of truth with the acerbic frowns of the entire community than purchase popularity by winking and silently smiling upon error. The sacrifice is too dear and too great, and we spurn the terms of the treaty. For our part, whatever the over-wise and politic may do, we will continue the warfare, because we conceive it our duty to combat error so long as it presents itself. If we make no converts, we may prevent others of the calling, of *easy attachment* to their profession from sliding into some of the various *isms*, whatever the prospect of gain or emolument.

But to what is owing the present successful efforts in empiricisms in the Queen city of the West? Have these over-cautious logicians found here an illustration of their *let alone* theory; or in other words, have the profession of that city been dealing out *pap* to them in a *mercurial* spoon? We know not. But it is, and has been, a matter of speculation with us why that city, the home of so many able and scientific men, present and past, should now be found to be the *hot bed* of quackery, and the point or center whence radiates more sickly and deathly empiricism than any one city of this wide-spread republic. It is looked upon as an opprobrium to that city, and would be regarded as *prima*

*facie* evidence that the school master has not been abroad and that the usual means of disseminating intelligence have been neglected, did we not know to the contrary.

Ohio has four regular schools, (enough in all conscience); it has as many ably conducted journals; it has a profession scattered all over the State, which, from the amount of talent and acquirement, is an honor to it, and yet in that State, perhaps, more than any other, quackery seems to thrive best. If they would but permit us to make a suggestion, it would be to bring the profession of the State together and consult in regard to the necessary means to put down this enemy to science and humanity. To accomplish this they must reform the public mind on the subject; and then the profession, to a man, should go to work. They should say to the people of that proud and populous city that the smiles of encouragement which they give to quackery, are exchanged for frowns from the people of other cities and States, because they would permit scientific enterprises to languish and die in their midst, and give vitality and vigor to "claptraps" and "mountebanks." What a humiliating spectacle! and how deeply chagrined must the regular profession be found to be when in association and contact with a bold and unprincipled band of mercenary charlatans. It is strange that a scientific physician will remain in that "Sodom," where he must be subjected to daily mortification and contumely. The truth is the profession of that State must make a united work of it—every man who has any regard for himself or his profession should do battle, and *do it manfully*, for it will not do to wait for quackery to cure itself, and all our aid would be given if near enough to render it of service.

It is true that we *have* moats in our own eyes. There are those in our ranks whose dishonorable conduct brings reproach upon us all. It is true that there are those, who, if they possessed the power would, actuated by a reckless vandalism, drag down the dignity of the profession and trample it under their feet with a triumphant gratification, so that by such a sacrifice, their own selfish ends may be accomplished, or their low acts prevented from being brought under the law of retribution. We *know* we have *Judases* and *Arnolds*, who would stop at no "nice extreme," in the effort to bolster up their own inherent rottenness and save them from the effects of that moral gravitating law which would bring them to their level sooner or later. Truth, honor, honesty, and virtue, must be sacrificed to conceal their baseness, and the standing and reputation of those "whose shoe strings they are unworthy to unloose," must be assailed and withered, that they may make room for them to gratify their own wicked designs and mercenary aims.

Such men do the profession incalculable injury, because they lower its standard in the estimation of the public and weaken the influence which it is capable of exerting in society. This constitutes in part the *pabulum* for empiricism, for it steps in and seizes the propitious moment in which to make a foothold.

It is therefore as much our duty to condemn these openly, as to oppose the *bold* empiric, because they have greater capacities for mischief. To breathe life into such, is as criminal on our part as to endorse the conduct of those "who slander, abuse, vilify, and cheat, the regular profession in every way they can devise," and like them "get buncombe reports of cases" which never occurred.

We ask the Medical Journals to look to this matter, for we have been made aware through other channels that the profession in that city are indeed unfairly dealt with in this particular. In their warfare they should be generously aided, rather than to give their enemies a weapon to hang *in terrorum* over their heads. It is in this way that worthy efforts can be thwarted, and the spirit of emulation stifled. We should endorse no man, nor combination of men, whose design is to prostrate an honest enterprise. In this work of reform, we trust the *Lancet* will join. While engaged in their own locality in a contest which is attended with mortification, the *Lancet* should remember that there are troubles elsewhere arising out of the workings of remorseless, corrupt, and designing individuals who are plotting more mischief and injury to the profession within, than can be accomplished by four-fold the number of open, avowed enemies from without. At the proper time and in the proper place, we will hold some of these traitors to their profession as high as Haman, as objects of scorn by the profession.

